# Differences in the Expression of TLR-2, NOD2, and NF-κB in Placenta Between Twins

**DOI:** 10.1007/s00005-018-0514-x

**Published:** 2018-05-23

**Authors:** Łukasz Szylberg, Magdalena Bodnar, Anna Lebioda, Patrycja Krepska, Adam Kowalewski, Grzegorz Bręborowicz, Andrzej Marszałek

**Affiliations:** 1 Chair and Department of Clinical Pathomorphology, Collegium Medicum in Bydgoszcz, Nicolaus Copernicus University in Toruń, Bydgoszcz, Poland; 2 Department and Clinic of Perinatology and Gynecology, Poznań University of Medical Sciences, Poznan, Poland; 3 Department of Obstetrics and Gynecology, Collegium Medicum in Bydgoszcz, Nicolaus Copernicus University in Toruń, Bydgoszcz, Poland; 4 Chair and Department of Oncologic Pathology and Prophylactics, Greater Poland Cancer Center, Poznań University of Medical Sciences and Department of Oncologic Pathology, Poznan, Poland; 5 Department of Pathomorphology, Military Clinical Hospital, Bydgoszcz, Poland

**Keywords:** Placenta, Immunology, Twins, NOD2, TLR-2, NF-κB

## Abstract

Dizygotic twins share the same type of genetic relationship as non-twin siblings. Whereas monozygotic (MZ) twins are considered to have identical genetic material, they still differ. There is a number of reasons for early MZ twin discordance, including differences in the in utero environment, stochasticity, genetic mosaicism, and epigenetic factors. During gestation, the efficient innate immune system is of utmost importance. Our study was based on immunohistochemical evaluation of the differences in innate immune protein expression (TLR-2, NOD2, and NF-κB) in the 95 placentas between twins. Our study revealed statistical significant differences between diamniotic–dichorionic and monoamniotic–dichorionic twins. Monoamniotic–monochorionic twins exhibited no significant differences in protein expressions. To identify epigenetic factors causing the differences between twins, we made a series of comparisons with clinical data. The study revealed more cases with infections, miscarriages, in vitro fertilization, and premature rupture of membranes within the group with higher differences level of NF-κB, NOD2 and TLR-2 between twins. In case of twin-to-twin transfusion syndrome, there were no significant differences in innate immune protein expressions between twins. These results show that dissimilar genetic material and separate in utero environment promote discordance in innate immune protein expressions between twins. Moreover, additional blood flow between twins may be favorable in life-threatening conditions ensuring similar microenvironment.

## Introduction

In recent years, the wide availability of assisted reproductive technologies has caused a rapid increase in number of multiple pregnancies. Twin pregnancies naturally occur with a frequency of 1:80 births but new opportunities have caused an increase in number of twin pregnancies by 47%, while triplet pregnancies have increased by 37% (Fauque et al. 2010).

Dizygotic (DZ) twins share the same type of genetic relationship as non-twin siblings (Titlestad et al. 2002). Whereas monozygotic (MZ) twins are considered to have identical genetic material, they still differ. Phenotypic discordance in MZ twins has traditionally been ascribed to non-shared environmental factors acting after birth; however, recent data indicate that this explanation is far too simple. There is a number of reasons for early MZ twin discordance, including differences in the in utero environment, stochasticity, genetic mosaicism, and epigenetic factors (Czyz et al. 2012).

Early embryonic development relies on properly functioning epigenetic modifications that mediate normal growth, cell differentiation, and morphogenesis (Inbar-Feigenberg et al. 2013; Nelissen et al. 2011). Likewise, normal placental development requires appropriate regulation of gene expression (Roifman et al. 2016). During gestation, the efficient innate immune system is of utmost importance. It constitutes the first line of defense against invading pathogens and plays a key role in maintaining the proper implantation of placenta (Loke and King 2000).

Innate immune cells express numerous receptors with Toll-like receptors (TLRs) as the main representative. TLRs bind and become activated by various ligands (Xie et al. 2010). TLR-2 is characterized by the widest specificity, detecting lipoteichoic acid of Gram-positive bacteria, lipopolysaccharide, peptidoglycan, fungal zymosan, and multiple lipoproteins (Basith et al. 2011; Good et al. 2012). When bounded to the specific ligand for TLR, leucine-rich repeat domain triggers two signaling pathways (Ayyar et al. 2007). One pathway depends on adapter molecule myeloid differentiation primary response protein 88 (MyD88), while the second is associated with NOD2 (Beutler et al. 2006; Kumar et al. 2009). The signal transduction pathways, in consequence, activate the nuclear factor kappa-light-chain-enhancer of activated B cells (NF-κB). Active NF-κB penetrates into the nucleus and induces expression of cytokines, adhesion molecules, and growth regulators (Takeda et al. 2003). Nod-like receptors (e.g., NOD2) are intracellular sensors of pathogen-associated molecular patterns, which together with TLRs regulate the inflammatory and apoptotic response.

Aiming to check if the twins differ in this area, we performed a comparative study of expressions of innate immune proteins (TLR-2, NOD2, and NF-κB) in twins’ placentas.

## Materials and Methods

The study was performed on 95 pairs of twins. After birth, the following tissues were taken (from both twins, in a repeatable manner): umbilical cord, placenta, and fetal membranes.

All of the collected tissue sections were processed according to standard diagnostic protocol in force in Department of Clinical Pathomorphology. First, collected tissue sections were fixed in 10% buffered formalin for 24 h in room temperature; after fixation, the sections were dehydrated in ethyl alcohols 80–99.8%, cleared in xylenes (I–IV), and embedded in paraffin.

### Immunohistochemistry

After preliminary evaluation of tissue sections according to hematoxylin and eosin staining, performed by two independent pathologists, the material from placentas was selected for immunohistochemical studies.

The paraffin tissue blocks were cutted into 4-µm-thick paraffin tissue sections and placed on extra adhesive slides (SuperFrostPlus, Thermo Scientific, Braunschweig, Germany). The proper immunohistochemical staining was followed by a series of positive and negative control reactions. The positive control reactions were performed on model tissue sections selected according to reference sources (The Human Protein Atlas), and from manufacture antibodies’ datasheets, whereas the presence of the analyzed antigens was established. For TLR-2, the positive control tissue was spleen, and the expression was evaluated in macrophages; for NOD2, the positive control tissue was the appendix, and the expression was evaluated in monocytes/macrophages and T lymphocytes. For NF-κB, the positive control tissue was colon cancer, and the expression was evaluated in cancer cells. The negative control was performed on additional control tissue sections by substituting the primary antibody with 1% solution of bovine serum albumin (BSA) in phosphate-buffered saline (PBS).

For the immunohistochemical staining, we used protocol described elsewhere (Bodnar et al. 2013). The immunohistochemical studies were performed using, respectively, rabbit polyclonal antibody against TLR-2 (ab24192, ABCAM, Cambridge, UK), rabbit polyclonal anti-NOD2 (PRS2511, Sigma-Aldrich, Poznan, Poland), rabbit polyclonal anti-NF-κB p65 (ab31481, ABCAM, UK). In brief, tissue sections were dewaxed in a series of xylenes and rehydrated in a series of ethyl alcohol (99.8–80%). Epitopes were unmasked in PT-Link (Dako, Denmark) using Epitope Retrieval Solution high-pH. Subsequently, the activity of endogenous peroxidase was blocked by 3% H_2_O_2_ for 10 min, and the non-specific antibody binding was blocked by 5% BSA in PBS. The incubation with primary antibodies was performed: for TLR-2 for 30 min in 37 °C, at the 1:400 dilution, for NOD2 for 30 min in 37 °C at the 1:500 dilution, and for NF-κBp65 for 16 h in 4 °C, with the 1:200 dilution. Finally, tissue sections were incubated with Anti Mouse/Anti Rabbit EnVision FLEX-HRP (Dako), for 20 min in 37 °C, and the antigen–antibody complex was localized using 3–3′diaminobenzidine (DAB) as a chromogen. The brown reaction product was observed in spite of the presence of antigen. At the end, the tissue sections were counterstained with hematoxylin, dehydrated, incubated in a series of xylenes, and mounted.

### Measurement of Protein Expression–Morphometric Principles

The pathologists who were evaluating the immunohistochemical expression of examined antigens worked independently, and they were blinded from the patients’ clinical, as well as other data. The protein expression was evaluated using light microscope ECLIPSE E800 (Nikon Instruments Europe, Amsterdam, Netherlands) with CD camera (Nicon Digital DS-5Mc, Germany) at × 20 original objective magnification. Using the NIS Elements 3.0 Software, the microphotographs were taken. To evaluate the immunohistochemical staining, automatic morphometric analyses, described and used in previous papers of the authors, were performed (Bodnar et al. 2014).

In this project we studied two parameters. The first parameter was the localization of immunohistochemical protein expression, which was specified in detail by two pathologists. The second parameter was the ratio between the size of the area expressing the given antigens, and the intensity of antigen expression performed by graphical software ImageJ ver. 1.46.

First, because the expression of analyzed proteins was homogenous in all tissue area, three representative microphotographs of each immunohistochemical expression in each case were taken. In total, 570 microphotographs for each studied protein (95 twin pairs × 3) were evaluated.

Taken images were modified using ImageJ functions, such as “Color deconvolution” and “Threshold function”. “Analyze and measure” software function was used as the last step. The results were expressed as the ratio of the positively stained area and the protein expression intensity (scale: 0–255), within syncytio and cytotrophoblast of the villi, as the median values. Moreover, the semiquantitative intensity of analyzed immunoexpression (weak/moderate/strong) was performed by two independent pathologists.

### Statistical Analysis

All statistical analyses were performed using Statistica version 10 (StatSoft) and Microsoft Excel 2007. The comparative studies were analyzed statistically using the nonparametric *U* Mann–Whitney and Kruskal–Wallis test. The *p* value < 0.05 was considered statistically significant. The expression values of analyzed proteins were presented as the median and average values.

## Results

### Patients’ Data

The clinical data were analyzed and systematized. There were 95 mothers of the twins aged 17–44 years (average: 29 years). At the moment of delivery, gestation time ranged 22–41 weeks (average: 35 weeks). The condition of newborns was evaluated by ten-point Apgar score (average: 8). There were 91 male and 99 female newborns. Birth weight ranged 280–3920 g (average: 2102 g). The type of pregnancy was recorded in 75 cases. Forty-nine pregnancies were diamniotic–dichorionic (Di–Di), 29 were monoamniotic–dichorionic (Mo–Di), and three monoamniotic–monochorionic (Mo–Mo). Whereas in 17 cases data on the type of preganancy could not be obtained. Moreover, it was found that 24 twins were MZ, whereas 23 were DZ. In other cases data concerning the zygosity of the twins could not be obtained.

Additional critical clinical data were as follows: infection from group B *Streptococcus* (GBS) was recorded in 23 cases, gestational diabetes mellitus (GDM) was diagnosed in 10 cases, premature rupture of membranes (PROM) occurred in 12 cases, past miscarriage (PM) was recorded in 10 cases, in vitro fertilization (IVF) was used in 10 cases, and twin-to-twin transfusion syndrome (TTTS) was diagnosed in four cases.

### NOD2, TLR-2, and NF-κB Expression

**Fig. 1:**
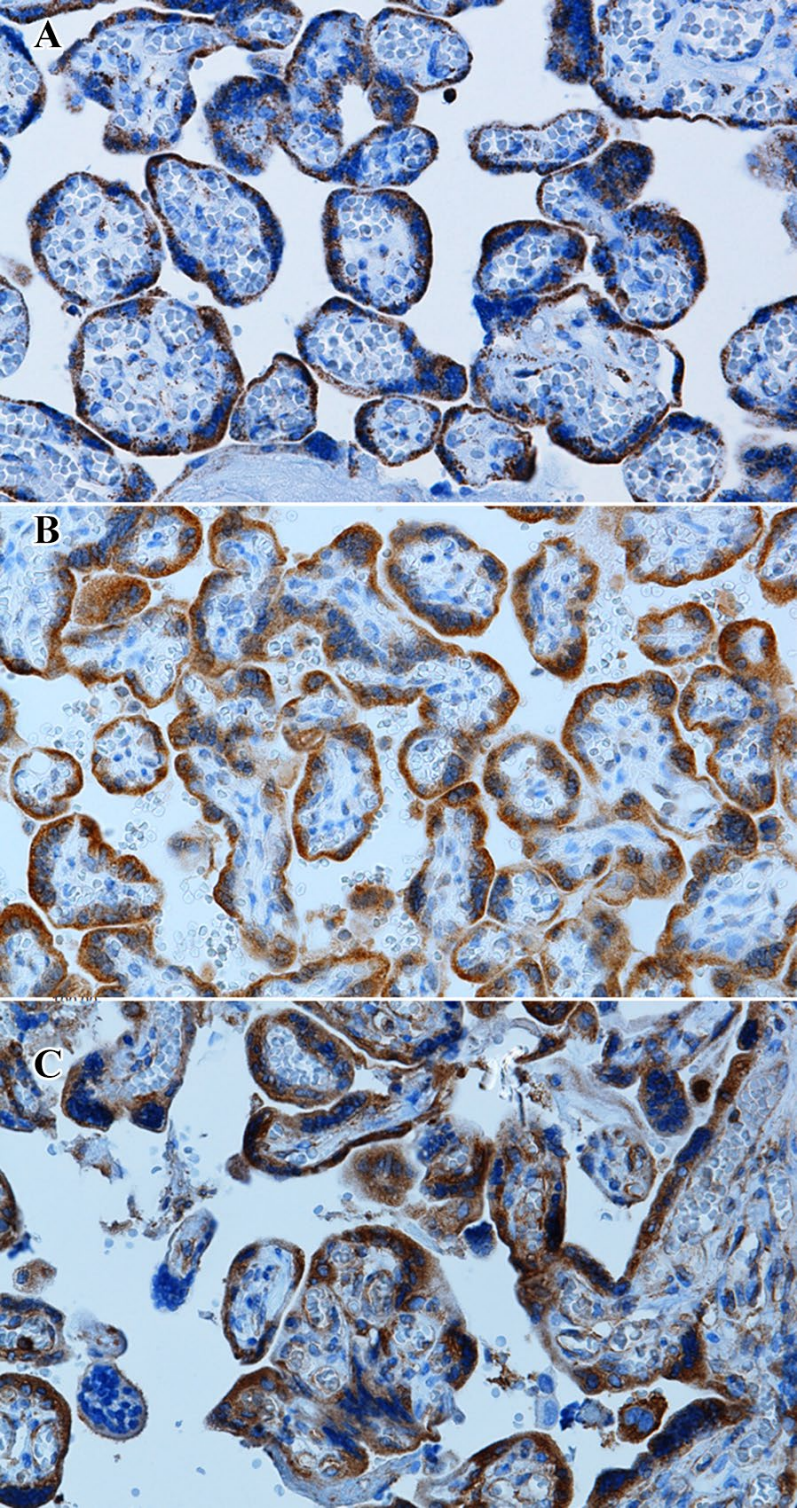
Representative microphotograph of immunohistochemical staining. The primary objective magnification × 20. TLR-2 (**a**); NOD2 (**b**); NF NF-κB (**c**)

The study revealed the cytoplasmic expression of NOD2 and TLR-2 in both cyto- and syncytiotrophoblast of the placenta. The NF-κB expression was found in cytoplasm and nucleus in both cyto- and syncytiotrophoblast of the placenta (Fig. 1).

In both cyto- and syncytiotrophoblast of the placenta, the expression level of NOD2 and NF-κB was moderate, while in some cases it was high. The median expression of NOD2 and NF-κB was 144.2 (SD: 73.21) and 106.17 (SD: 60.07), respectively. TLR-2 protein revealed low expression, median 19.79 (SD: 28.21).

### Differences in the Expression of Proteins Between Twins

**Fig. 2:**
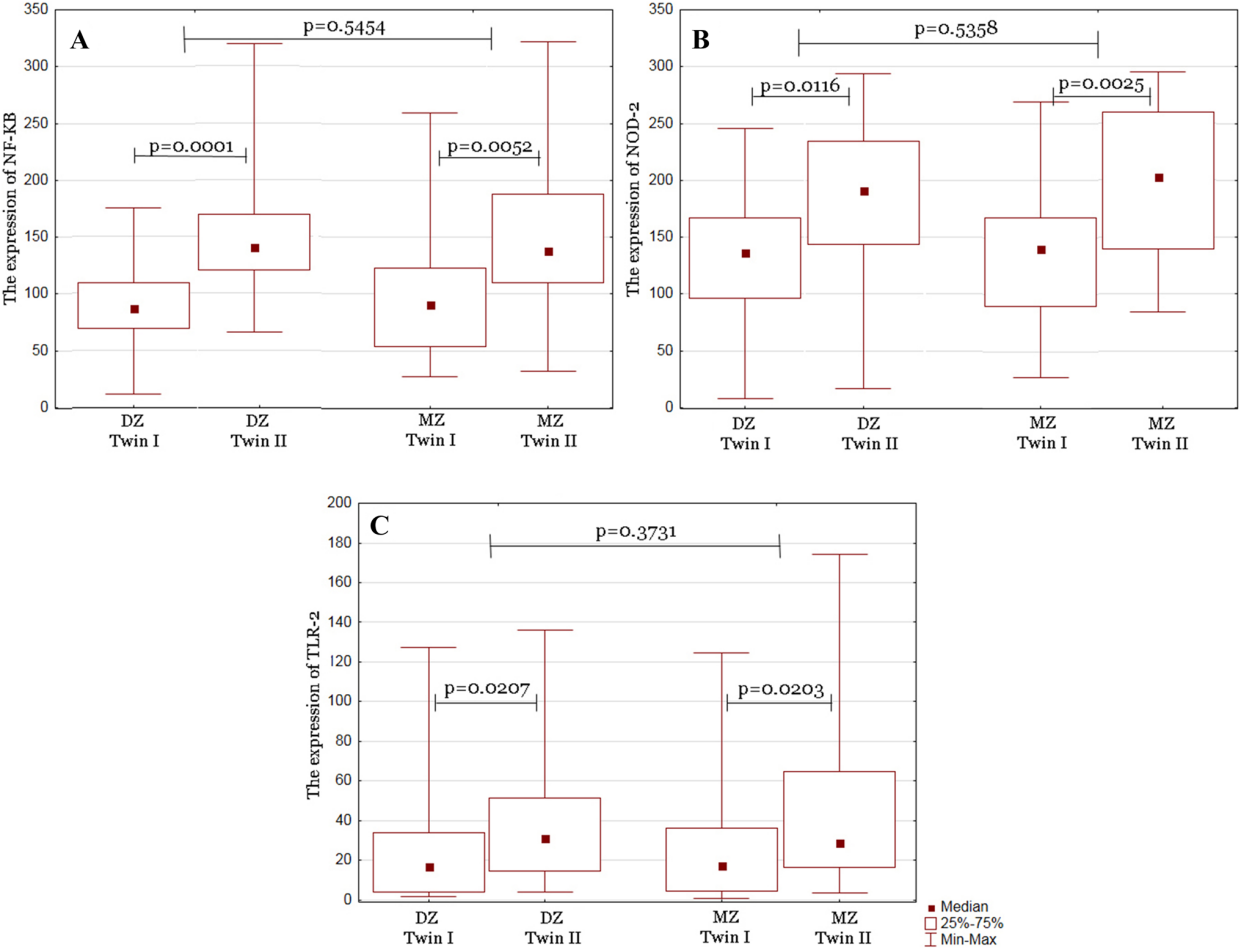
A box-plot of the differences between monozygotic (MZ) and dizygotic (DZ) twins in: NF-κB expression (**a**), NOD2 expression (**b**), and TLR-2 expression (**c**)

**Fig. 3:**
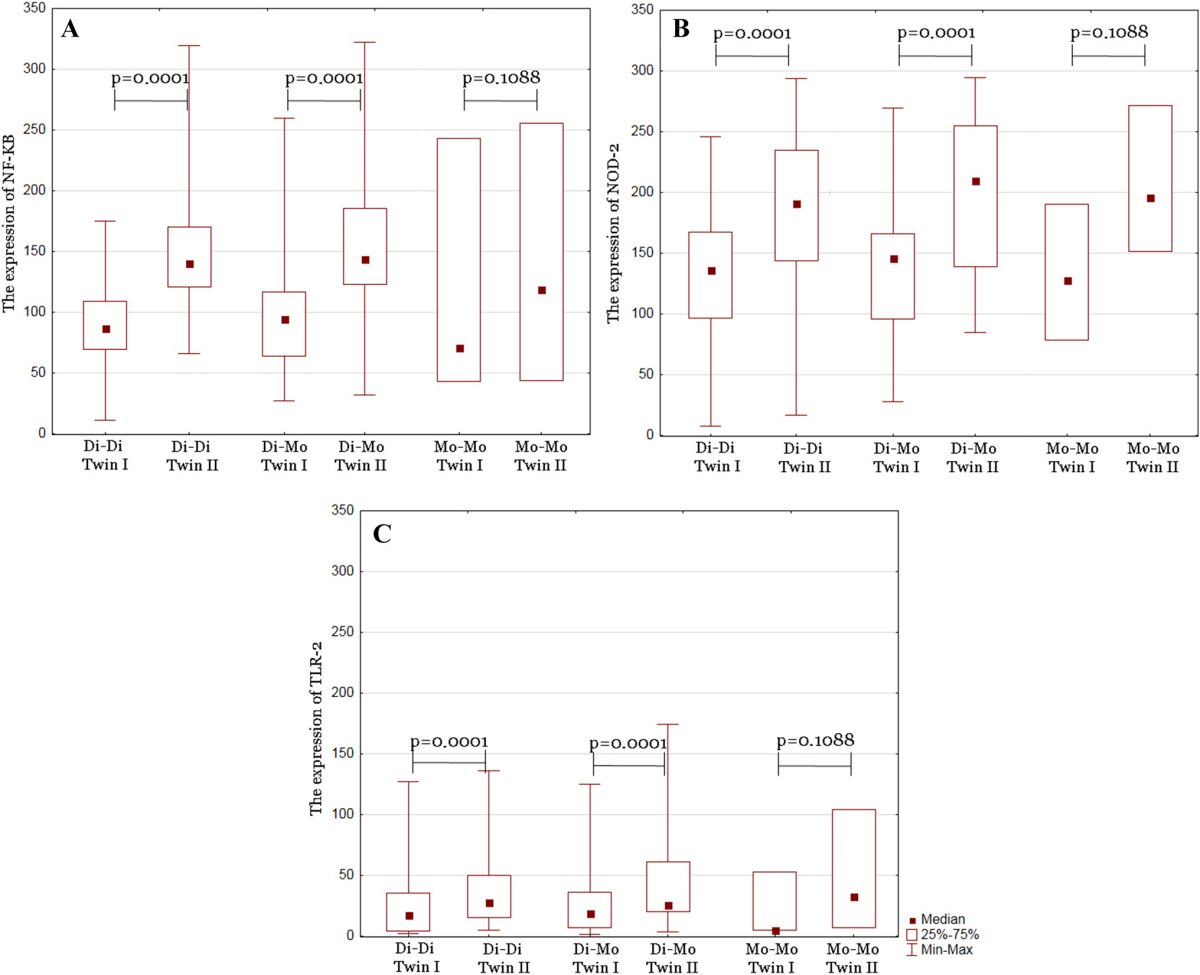
A box-plot of the differences between twins in diamniotic–dichorionic (Di–Di), monoamniotic–dichorionic (Mo–Di), and monoamniotic–monochorionic (Mo–Mo) groups in: NF-κB expression (**a**), NOD2 expression (**b**), and TLR-2 expression (**c**)

We compared the differences between median expression levels of TLR-2, NF-κB, and NOD2 in groups of MZ, DZ, Di–Di, Mo–Di, and Mo–Mo twins. Statistical analysis revealed statistical significance with prominent difference level of TLR-2, NF-κB, and NOD2 between twins in groups of MZ, DZ, Di–Di, and Mo–Di. The only group which did not reach statistical significance was Mo–Mo (Figs. 2, 3). Differences in NF-κB, NOD2, and TLR-2 expression between twins are summarized in Table 1. To identify factors affecting the differences between twins, we correlated the results with clinical data. We compared the differences between median expression levels of TLR-2, NF-κB, and NOD2 in groups of GBS, PM, IVF, PROM, and TTTS. Statistical analysis revealed statistical significance with observable difference level of TLR-2, NF-κB, and NOD2 between twins in groups of GBS, PM, IVF, and PROM. Study did not reveal any statistical significant correlation between twins and TTTS. These results are presented in Table 2.

**Table 1:** Summarized differences in NF-κB, NOD2, and TLR-2 expression between twins

Clinical data	Protein	Average ± SD Twin I	Average ± SD Twin II	Statistical significance
Di–Di	NF-κBB	86 ± 36	140 ± 57	*p* = 0.001
	NOD2	136 ± 68	190 ± 76	*p* = 0.001
	TLR-2	17 ± 29	31 ± 30	*p* = 0.001
Mo–Di	NF-κB	94 ± 53	143 ± 66	*p* = 0.001
	NOD2	145 ± 60	209 ± 70	*p* = 0.001
	TLR-2	18 ± 29	25 ± 43	*p* = 0.001
Mo–Mo	NF-κB	70 ± 108	118 ± 107	ns
	NOD2	127 ± 56	195 ± 61	ns
	TLR-2	4 ± 27	32 ± 50	ns
MZ	NF-κB	90 ± 59	138 ± 69	*p* = 0.005
	NOD2	140 ± 58	202 ± 68	*p* = 0.002
	TLR-2	18 ± 29	29 ± 43	*p* = 0.019
DZ	NF-κB	86 ± 36	140 ± 58	*p* = 0.001
	NOD2	136 ± 68	190 ± 76	*p* = 0.011
	TLR-2	17 ± 29	27 ± 29	*p* = 0.019

*Di*–*Di* diamniotic–dichorionic, *Mo*–*Di* monoamniotic–dichorionic, *Mo*–*Mo* monoamniotic–monochorionic, *MZ* monozygotic, *DZ* dizygotic, *ns* not significant

**Table 2:** Summarized differences in NF-κB, NOD2, and TLR-2 expression between twins

Clinical data	Protein	Average ± SD Twin I	Average ± SD Twin II	Statistical significance
GBS	NF-κB	106 ± 16	137 ± 39	*p* = 0.001
	NOD2	164 ± 43	250 ± 36	*p* = 0.001
	TLR-2	13 ± 28	28 ± 34	*p* = 0.001
PM	NF-κB	100 ± 33	125 ± 58	*p* = 0.018
	NOD2	107 ± 56	191 ± 76	*p* = 0.016
	TLR-2	23 ± 20	52 ± 26	*p* = 0.017
IVF	NF-κB	78 ± 15	135 ± 81	*p* = 0.028
	NOD2	157 ± 37	211 ± 44	*p* = 0.027
	TLR-2	7 ± 24	17 ± 16	*p* = 0.028
PROM	NF-κB	67 ± 23	126 ± 75	*p* = 0.012
	NOD2	124 ± 71	185 ± 71	*p* = 0.011
	TLR-2	15 ± 17	39 ± 36	*p* = 0.022
GDM	NF-κB	99 ± 17	150 ± 39	*p* = 0.028
	NOD2	158 ± 43	242 ± 36	*p* = 0.028
	TLR-2	25 ± 28	42 ± 34	*p* = 0.028
TTTS	NF-κB	127 ± 20	160 ± 43	ns
	NOD2	146 ± 13	244 ± 45	ns
	TLR-2	10 ± 9	16 ± 8	ns

*GBS* group B streptococcus infection, *PM* past miscarriage, *IVF* in vitro fertilization, *PROM* premature rupture of membranes, *TTTS* twin-to-twin transfusion syndrome, *GDM* gestational diabetes mellitus, *ns* not significant

## Discussion

Twins do not necessarily share in utero microenvironment. Uneven blood supply is the most influential among environmental factors that cause early discordance between twins (Czyz et al. 2012). There is a variety of possible placental dysfunctions including infarcts, stem vessel thrombosis, velamentous insertion of the cord, single umbilical artery, or placental abruption. Each condition may potentially result in intrauterine growth restriction (IUGR). IUGR, in turn, can lead to discordance in birth weight, intelligence, language comprehension and expression, motor performance, balance coordination, and visual-motor perception. Uneven blood supply especially affects monochorionic (MC) twins that are seven times more likely to develop congenital heart disease (Czyz et al. 2012; Springer et al. 2014). In case of TTTS, the risk is increased even further (Lewi et al. 2010).

Random movements of molecules and the complexity of their interactions result in transcriptional and translational stochasticity (Zernicka-Goetz and Huang 2010). Unequal blastomere allocation may account for diminished ability of the smaller embryo to acquire an adequate share of an MC placenta, and in rare and extreme cases resulting in twin reversed arterial perfusion sequence and parasitic conjoined twins (Machin 2009).

Phenotypic discordance between MZ twins can be also attributed to de novo events such as chromosomal mosaicism (e.g., the discordant karyotypes mos 47, XX; + 21/46, XX; and 46, XX) (Choi et al. 2013), differing copy number variations (Breckpot et al. 2012) and distinct single nucleotides (Castellani et al. 2015). Postzygotic point mutations have been found to be the source of MZ twin discordance in oral-facial-digital syndrome type 1, neurofibromatosis type 1, Van der Woude syndrome, Joubert syndrome, and Darier’s disease (Czyz et al. 2012). Moreover, unequal exchange of cells during gestation might lead to discordant fetomaternal microchimerism (Gringras and Chen 2001).

Maintaining the proper implantation of placenta by innate immune system can be attributed to epigenetic processes as well as to genetic modifications (Guleria and Pollard 2000; Loke and King 2000). Genetic modifications have a greater impact on developing fetus, but occur much less often. The main epigenetic marks include DNA methylation, histone modifications, and non-coding RNAs (Nelissen et al. 2011). The studies performed on cells collected from twins revealed that about one-third of the MZ twins demonstrate epigenetic differences in DNA methylation and histone modification (Fraga et al. 2005). These findings support our studies which demonstrate the differences in innate immune protein expression in one-third of the twins’ pairs.

Placental epigenetic dysregulations may cause abnormalities in trophoblast differentiation, angiogenesis, and endocrine signaling (Nelissen et al. 2011), which, in turn, can be detrimental for both mother and child (Inbar-Feigenberg et al. 2013). Mosaicism in epigenetic alterations has been described in MZ twins (Czyz et al. 2012; Galetzka et al. 2012). Roifman et al. (2016) proved that placental epigenetic dysregulation underlies the aberrant gene expression in the pathophysiology of IUGR.

Our study was based on evaluation of the differences in innate immune protein expression in the placenta between twins. The greatest differences were noted between Di–Di twins. Among Mo–Di twins, the differences were smaller but still significant. Mo–Mo twins exhibited no significant differences in protein expressions. These results show that dissimilar genetic material and separate in utero environment promote discordance in innate immune protein expressions between twins.

Expressions of NOD2 and NF-κB between twins were significantly more discordant than expression of TLR-2. Presumably, as TLR-2 plays a critical role in the innate immunity, the level of its expression must maintain within a narrow range.

To identify epigenetic factors causing the differences between twins, we made a series of comparisons with clinical data. The factors that were taken into consideration included a history of infection during pregnancy (GBS), GDM, miscarriage in the past (PM), IVF, PROM, and TTTS. The study revealed more cases with infections, miscarriages, IVF, and PROM within the group with higher differences level of NF-κB and TLR-2 between twins.

Infections are strongly associated with innate immune response mechanisms. Clinical studies demonstrated a strong correlation between intrauterine infection and certain pregnancy complications, such as pre-eclampsia, IUGR, and preterm delivery (Arechavaleta-Velasco et al. 2008; Goldenberg et al. 2000). In the five most studied activation pathways, numerous genes related to immune regulators undergo intensified changes (Mikheev et al. 2008; Winn et al. 2007). In neonates, GBS is one of the most important pathogens in the early onset neonatal infection (Stoll et al. 2011). Few studies have demonstrated the substantial role of TLR-2 in GBS infection (Draper et al. 2006; Henneke et al. 2008). Bacterial lipoproteins constitute the dominant TLR-2 activating molecules from GBS. During sublethal infection, bacterial lipoproteins activate local defense to ensure immediate elimination of GBS. If this rapid TLR-2-mediated response is insufficient, GBS will further disseminate and generalize inflammation, multiorgan failure, and even death may potentially ensue (Henneke et al. 2008). Considering the specific and complex role of placenta during the modulation of immune response and its exposure to environmental factors, it is not surprising that particular immune regulators are among the most variably methylated genes. The GBS infection might entail considerable differences in innate protein expression between twins because of distinct exposures to the pathogen. Discordant GBS infections are mostly reported in Di–Di twins; more frequently they occur in the sac nearest the cervix because of the ascending route of transmission (Mazor et al. 1996; Usta et al. 2002). The spatial orientation of the sac may play a role in determining the risk of infection of the second sac. If both sacs have a section adjacent to the cervix, the likelihood of their infection is expected to be greater than if only one sac fills the entire lower uterine segment (Usta et al. 2002). Presumably, placenta of the Twin A (usually designated as the first born) is characterized by higher expression of innate immune proteins comparing to placenta of the Twin B.

GDM is the most prevalent metabolic disorder during pregnancy, causing considerable morbidity and long-term complications for mother and child (DeSisto et al. 2014). GDM alters the expression of *AGER, CCL2*, and *CCL8* which are associated with generalized innate immune response (Money et al. 2017). GDM also induces excessive inflammatory activation in neonatal monocytes via TLR-2 (Yanai et al. 2016). Placenta is sensitive to the hyperglycemic milieu and responses with various adaptive changes of the structure and function. Most placentas from GDM pregnancies present typical histological findings (Jarmuzek et al. 2015). Placentas from twin pregnancies complicated by GDM, however, have significantly lower rates of vascular malperfusion lesions, villous immaturity, and villitis as compared to singleton gestations. These findings suggest different alterations in placental development and function in twins vs. singleton pregnancies complicated with diabetes (Weiner et al. 2018). This is why explanation of divergent expressions of innate proteins in twins’ placentas in the course of GDM might be even more complex and remains to be elucidated.

Placentas of twins may differ in superficial and deep vascular anastomoses, which are common in monochorionic twins. Additional blood flow between twins may be favorable in life-threatening conditions ensuring similar microenvironment. In our study, we observed smaller differences in TLR-2, NOD2, and NF-κB expression between twins in monochorionic group, which confirms this concept. In case of TTTS, there were no significant differences in innate immune protein expressions between twins.

We found no proof that other factors influence the differences in expressions of TLR-2, NOD2, or NF-κB among twins.
